# Response to letters to the editor on ‘Reducing plastic in single-use central line insertion packs: a mixed methods observational study’

**DOI:** 10.1177/0310057X251387729

**Published:** 2025-12-30

**Authors:** Alexandra R Seville, Luise Kazda, Scott McAlister, Katy JL Bell

**Affiliations:** 1 The Children’s Hospital at Westmead, Paediatric Intensive Care; Westmead, Australia; 2 NSW Health, Climate Risk and Net Zero, St Leonards, Australia; 3 University of Canberra, HEAL Global Research Centre, Canberra, Australia; 4 The University of Sydney, Wiser Healthcare Research Collaboration, Sydney, Australia; 5 The Healthcare Carbon Lab, Department of Critical Care, University of Melbourne, Melbourne, Australia

We thank Dr Zazay, Dr Burmeister, and Dr Sirohiya for their interest in our paper and the opportunity to provide further context to our study.^1^ We agree with Dr Zazay and Dr Burmeister that there are substantive challenges to implementation of sustainable practice in healthcare delivery. Top-down interventions are needed at system level such as reform of procurement processes and energy supply, as well as bottom-up clinician-led initiatives like the one described in our paper. The onus to implement sustainable healthcare cannot solely be on individual clinicians; they need to be supported by policy reforms at multiple levels of government and in health services.

We agree with Dr Sirohiya that a procedural audit or direct observation of pack use is an important step before designing and implementing interventions. The idea for the intervention in our study came from observations that the lead author had made in her clinical practice of critical care nursing, on the significant waste associated with central venous catheter (CVC) insertions. These observations prompted the development of our research question and a scoping review,^2^ which informed study design and data collection for this project, conducted as part of the New South Wales (NSW) Net Zero Leads partnership pilot programme.^3^ We also collected data provided by the clinic product coordinator to audit pack usage before commencing the study.

Dr Sirohiya raises concerns about infection control, an issue noted by Dr Zazay and Dr Burmeister as also serving as a disincentive to reusables despite little evidence to support perceived safety issues there. Indeed, patient safety was a central consideration in our study and clinician perspectives reflected this. An intensive care physician noted: ‘[Reducing] plastic is important, but it should not be counterproductive to the purpose for which these packs were built, which was an emergency. Having the central line, all the things available’, (ID 18, intensive care, 24 years experience).

Clinicians recognised the theoretical risk of contamination from retrieving additional items, but perceived the actual risk to be minimal. One anaesthetist noted: ‘I think it’s [a] theoretical problem, but I think with care and particularly in theatres where you’ve got everything in a drawer next to you (. . .). It’s not an issue I worry about’ (ID 17, anaesthetics, 23 years experience). Infection control concerns are often cited as a barrier to implementing sustainable healthcare practices and the two may be perceived to be in conflict. However, an increasing number of studies have found that they may often be synergistic. For example, the 2025 ‘Gloves off!’ study by Wilkie-Miskin et al. highlights that the use of non-sterile gloves can be associated with poorer hand hygiene and that a reduction in glove use may actually lead to better infection control.^4^

We appreciate the observation that workflow impacts were not costed in our analysis. While we are cognisant of the time pressures in the critical care environment, our decision to omit this was informed by the proximity of stock trolleys and rooms to the operating environment at both hospitals studied. Clinicians reported minimal concern regarding access to omitted items, with one noting, ‘Everything else is stocked in here that you need’, (ID 15, intensive care nurse practitioner, 18 years experience), and another stating ‘That’s readily accessible to get more, it’s even in our bedside trolley. So if you really need it, you could grab another one’ (ID 15, intensive care nurse practitioner, 18 years experience). Stock rotation due to shortages—particularly evident during the COVID-19 pandemic—has required clinicians to adapt frequently. Our survey and interviews indicated minimal clinician concern about using a reduced pack.

In terms of our environmental benefit calculation, we are aware of the prior research on central line kits, with one of our team having performed the original research (cited by Dr Sirohiya) in 2012.^5^ As in that study, the quantified values used in the current study were ‘cradle to grave’, and included all parts of the life cycle including raw material extraction, manufacture, sterilisation, all transport including from the site of manufacture and for waste disposal, and end of life, which in Australia for clinical waste is steam sterilisation followed by landfill in a prescribed waste site. As we noted in the paper, this was modelled using the LCA software SimaPro, using the ecoinvent and AusLCI background LCA databases.

In terms of the scalability of our intervention, we agree that a concerted effort is needed to renegotiate contracts. In NSW, procurement for all public hospitals is centralised and changes to custom packs require coordination with suppliers and government. Emphasising the need to consider not only financial costs, but also carbon emissions, in procurement decisions may help to achieve the systemic and policy change required for broader implementation.

Finally, we agree that strategies aiming to change clinician behaviour and culture around waste are needed.^6^ Our findings may inform the design of such behaviour change intervention, by providing empirical evidence on each of the capability-opportunity-motivation-behaviour domains of the COM-B model of the behaviour change wheel. Co-designing interventions with clinicians as the end-users recognises the importance of their perspectives to the successful implementation of any subsequent interventions. Our work provides the important foundational knowledge that is needed to implement broader initiatives that promote environmentally sustainable healthcare in the intensive care unit and operating theatres, including preventing medical waste and overuse.^7^
